# Self-rated health and associated factors among older South Africans: evidence from the study on global ageing and adult health

**DOI:** 10.3402/gha.v6i0.19880

**Published:** 2013-02-06

**Authors:** Nancy Phaswana-Mafuya, Karl Peltzer, Witness Chirinda, Zamakayise Kose, Ebrahim Hoosain, Shandir Ramlagan, Cily Tabane, Adlai Davids

**Affiliations:** 1 HIV/AIDS/STI/and TB (HAST), Human Sciences Research Council, South Africa; 2 Office of the Deputy-Vice Chancellor, Nelson Mandela Metropolitan University, Port Elizabeth, South Africa; 3 School of Human and Community Development, University of the Witwatersrand, Johannesburg, South Africa; 4 Department of Social Work, School of Human and Community Development, University of the Witwatersrand, Johannesburg, South Africa

**Keywords:** adult health, ageing, self-reported health, disability, quality of life, SAGE, South Africa, WHODAS-II, WHOQoL, ADLs, IADLs

## Abstract

**Background:**

Population ageing has become significant in South African society, increasing the need to improve understandings of health and well-being among the aged.

**Objective:**

To describe the self-reported ratings of overall health and functioning, and to identify factors associated with self-rated health among older South Africans.

**Design:**

A national population-based cross-sectional survey, with a sample of 3,840 individuals aged 50 years and older, was completed in South Africa in 2008. Self-reported ratings of overall health and functioning were measured using a single self-reported health state covering nine health domains (used to generate the Study on Global Ageing and Adult Health (SAGE) composite health state score). Disability was measured using the World Health Organization Disability Assessment Schedule II (WHODAS-II) activities of daily living (ADLs), instrumental activities of daily living (IADLs), perceptions of well-being, and the World Health Organization Quality of Life index/metric (WHOQoL).

**Results:**

Overall, more than three quarters (76.8%) of adults rated their health as moderate or good. On balance, men reported very good or good health more often than women (*p*<0.001). Older people (aged 70 years and above) reported significantly poorer health status than those aged 50–59 (adjusted odds ratio (AOR) 1.52; 95% confidence interval (CI) 1.00–2.30). Indians and Blacks were significantly more likely to report poorer health status at (AOR=4.01; 95% CI 1.27–12.70) and (AOR=0.42; 95% CI 0.18–0.98; 30 p <0.045), respectively, compared to Whites. Respondents with primary education (AOR=1.83; 95% CI 1.19–2.80) and less than primary education (AOR=1.94; 95% CI 1.37–2.76) were more likely to report poorer health compared to those with secondary education. In terms of wealth status, those in low wealth quintile (AOR=2.02; 95% CI 1.14–3.57) and medium wealth quintile (AOR=1.47; 95% CI 1.01–2.13) were more likely to report poorer health status than those in high wealth quintile. Overall, the mean WHODAS-II score was 20%, suggesting a low level of disability. The mean WHOQoL score for females (Mean=51.5; SD=12.2) was comparable to that of males (Mean=49.1; SD=12.6).

**Conclusions:**

The depreciation in health and daily functioning with increasing age is likely to increase demand for health care and other services as people grow older. There is a need for regular monitoring of the health status of older people to provide public health agencies with the data they need to assess, protect, and promote the health and well-being of older people.

It is crucial to understand the health of older people given the fact that the world's population is rapidly ageing, and estimates show that this increase will continue (1, 2). Similar trends have been observed in South Africa which has the continent's highest percentage of older inhabitants −6.7% of the population was estimated to be 60 years or older in 2005 (3). In South Africa, the older population currently constitutes 7.7% (4). The growth in the numbers of older persons, combined with the exigencies of growing old, puts pressure on governments to respond and to provide for the needs of this population. In this regard, the United Nations Madrid International Plan of Action on Ageing (5) and the 2003 African Union Policy Framework and Plan of Action on Ageing (6) were developed to urge governments to take account of ageing and older populations.

Earlier studies have shown that self-rated health of older people typically deteriorates with increasing age (7–9). Despite women having higher life expectancy than men, older men have consistently reported better health than their female counterparts (10) even after adjustments for differences in demographic and socioeconomic factors. Older people without formal education, without a spouse, and with low socioeconomic status (SES) are significantly more likely to self-rate their health status as poor compared to their more educated, married, younger, and higher SES counterparts (10, 11).

Earlier studies have also shown that quality of life (QoL) deteriorates with age, and higher proportions of women reported poor QoL compared to their male counterparts (12, 13). These studies showed that older age group, lack of formal education, being single, and currently not working were significantly associated with lower QoL (10, 11, 13). Lower QoL was also associated with poorer self-rated health (14).

Further, previous studies found that the incidence of disability and impairment increases with age (11, 15, 16). Women generally reported poorer functionality than men (11). Functional problems were compounded, if the older person had no spouse, had lower education, was unemployed, and was economically unstable (13, 16). Disability and impairment were also associated with poorer self-rated health (17). These findings need to be further explored among elderly people in South Africa, where health research has historically been more heavily focused on younger age populations.

A number of smaller scale studies (18–20) were conducted. National level studies such as the 2003 World Health Survey and the 1998/2003 South African Demographic and Health Surveys (SADHS) had limited samples of individuals aged 50 years and above, and, thus, the data were inadequate to inform policy formulation and program implementation. As the South African population ages, there is an increasing need for valid and comparable data on the health and well-being of older adults. There is a need to build an evidence base that can be used in formulating policies and monitoring their impact. Against this background, the Study on Global Ageing and Adult Health (SAGE) was conducted in 2008 as the first nationally representative population-based study among older South Africans. This study highlights the health needs of elderly South Africans upon which strategies to enhance health service provision for Africa's older persons can be built (5). The study provides high quality baseline data, which improve the understanding of health and well-being of older adults and ageing in South Africa, needed to inform policy and program debates.

## Methods

### Sample and procedure

The Human Sciences Research Council (HSRC), the World Health Organization (WHO), and the National Department of Health (NDOH) conducted a national population-based cross-sectional study, namely SAGE. Data for SAGE were collected between March 2007 and September 2008 in South Africa among individuals aged 50 years and above. The SAGE sample design entailed a two-stage probability sample that yielded national and sub-national estimates to an acceptable precision at provincial level, by locality type (urban and rural) and race (including Black, Colored, Indian or Asian, and White). The target sample size for SAGE was 5,000 individuals aged 50 years and above. A total of 3,840 individuals aged 50 years or older agreed to participate in SAGE. This gave an individual response rate of 77%. The study was approved by the HSRC Research Ethics Committee and the NDOH.

### Measures

#### Sociodemographic characteristics

These included age, sex, education, race, religious affiliation, marital status, geolocality (divided into rural and urban), and wealth status.

#### Self-rated health

Overall, self-rated health status was based on respondents’ assessment of their current health status on a 5-point scale in response to the question: ‘In general, how would you rate your health today?’ Response categories were: very good, good, moderate, bad, and very bad. The WHO's approach to measuring health states uses multiple domains of health that explain 80% of the variance in an individual's health (21). This approach provides scalable levels of health and the ability to decompose a single score into meaningful components. Respondents also rated their health on nine domains: affect, mobility, sleep and energy, cognition, interpersonal activities, vision, self-care, pain, and breathing (22). The SAGE composite health score was derived from 16 responses, two questions for each domain, using a Rasch partial credit model of Item Response Theory (23) that served to generate a composite health-state score (24, 25). A Chi-square goodness of fit statistics was calculated to determine how well each item contributed to a common global health measurement. The calibration for each of the health items was taken into account, and the raw scores were transformed into a continuous cardinal scale, where a score of 0 represents the worst health and a maximum score of 100 represents the best health.

#### Difficulty in carrying out work or household activities

The time period was specified as ‘In the last 30 days’, and the respondents were asked to provide an average of good and bad days. This indicator is intended to measure the impact of health on a person's functioning.

#### Disability

The 12-item version of WHO Disability Assessment Schedule (WHODAS-II) (www.who.int/icidh/whodas/) was used to measure health, functioning, and disability. WHODAS II evaluates six domains (two items per domain) of day-to-day functioning in the last 30 days – understanding and communicating, getting around, self-care, getting along with people, life activities, and participation in society (15). It contains many of the most commonly asked activities of daily living (ADLs) and instrumental activities of daily living (IADLs) questions, with response categories that provide an estimate of severity of disability through asking about the level of difficulty with each activity. Levels range from ‘no difficulty at all’ to ‘extreme difficulty/cannot do’. ADLs are basic daily self-care activities that support survival, including eating, bathing, and toileting. They usually assess the need for help with personal care activities, such as eating, bathing, and dressing. An individual's ability to perform ADLs is typically considered normal functional status, with an inability to perform ADLs suggesting disability. IADLs are indicators of functional well-being that measure the ability to perform more complex tasks, such as heavy or light housework, laundry, preparing meals, shopping for groceries, getting around outside, travelling, managing money, and using a telephone. Results from the 12 items were summed to get an overall WHODAS-II score, which was then transformed to a 0 (no disability) to 100 (high disability) scale.

#### Subjective well-being and *QoL*

Subjective well-being includes a person's overall appraisal of his or her life (global well-being) and affective state (hedonic well-being), and it is an important aspect of older people's health (26). For this study, subjective well-being and QoL were measured using the eight-item World Health Organization Quality of Life (WHOQoL) instruments. The WHOQOL instrument contains a set of international, cross-cultural comparable tools used to assess QoL and provides a measure of the evaluative component of well-being (27). The instrument ranges from 0 to 100 and was evaluated by responses to questions on overall life satisfaction and specific aspects of life. It also used two questions in each of the four broad domains: physical, psychological, social, and environmental domains (28). Results from the eight items were summed up to get an overall WHOQOL score, which was then transformed to a 0–100 scale, with lower scores indicating a better QoL. Besides overall satisfaction, SAGE also asked whether an older person was satisfied with a wide range of life aspects – health, oneself, ability to perform ADLs, personal relationships, and conditions of living space.

#### Experience of happiness

SAGE used the Day Reconstruction Method (DRM) to measure the experienced happiness component (29, 30). The methodology of the DRM entails asking participants to think about the preceding day, break it down into episodes, and then describe each episode in terms of the activity engaged in, the accompanying positive and negative emotions, the amount of control the respondent had over the activity, and the context in which the activity was carried out.

#### Household wealth

It was measured from possession of assets, such as television, radio, and fridge, as well as access to amenities, such as electricity, water, and toilet facilities. Principal component analysis was used to derive household wealth scores, which were later categorized into quintiles.

### Data analysis

Data were captured onto United States Census Bureau's Census and Survey Processing System (CSPro) version 3.0.1 and converted into STATA version 10 (Stata Corporation, College Station, Texas, USA) for statistical analyses. It was weighted using post-stratified individual probability weights based on the selection probability at each sampling stage. Individual weights were post-stratified by province, sex, and age-group, according to the 2009 Mid-Year population estimates from Statistics South Africa (31). Associations between key outcomes of self-reported health and sociodemographic, social, and health variables were assessed using odds ratios (OR). Unconditional multivariable logistic regression was used for evaluation of the association of explanatory variables, with a key outcome (poor self-rated health). A dichotomous measure was created for self-reported health, where responses ‘very good’, ‘good’, or ‘moderate’ were grouped into one category, that is, 0, and responses ‘bad’ or ‘very bad’ were grouped into a secondary category, that is, 1.

All variables statistically significant at the *p*<0.05 level in univariate analyses were included in the multivariable models. In the analysis, weighted percentages have been reported. The reported sample size refers to the sample that was asked the target question. The two-sided 95% CIs have been reported to indicate significance levels and were adjusted for the multi-stage stratified cluster sample design of the study. Interaction between predictor variables was also examined, and it was found that none of the variables had a variance inflation factor (VIF) value above 2.5.

## Results

### Sample characteristics

Slightly more than half (55.9%) of the respondents were women. The dominant racial group was African Black (74%), and almost half (49.9%) were between 50 and 59 years. The educational level of most participants (71.6%) was lower than secondary school education, and almost two-thirds (64.9%) lived in an urban area. Overall, there were no major wealth differentials in this sample (Table 1).

**Table 1:** Sociodemographic characteristics of adults aged 50 years or older, per cent distribution by sex

Sociodemographic characteristics	Men (*N*=1,690)	Women (*N*=2,146)	Total (*N*=3,836) *n* (%)
Age group (years)			
50–59	52.2	48.1	1,914 (49.9)
60–69	30.5	30.7	1,174 (30.6)
70–79	11.4	16.0	536 (14.0)
80+	5.8	5.3	212 (5.5)
Ethnic background			
African/Black	73.7	74	2,337 (73.9)
White	10.7	8.3	293 (9.3)
Colored	11.8	13.5	406 (12.8)
Indian/Asian	3.7	3.9	121 (3.8)
Unknown	0	0.1	2 (0.1)
Other	0.1	0.1	3 (0.1)
Residence			
Urban	65.8	64.1	2,489 (64.9)
Rural	34.2	35.9	1348 (35.1)
Marital status			
Never married	8.3	18.6	539 (14.1)
Currently married	71.9	31.9	1901 (49.5)
Cohabiting	7.4	3.8	207 (5.4)
Separated or divorced	3.8	7.4	224 (5.8)
Widowed	7.6	35.9	900 (23.5)
Education			
No formal education	22.2	27.1	774 (25.2)
Less than primary education	25.2	23.2	738 (24.0)
Primary school completed	22.0	22.7	688 (22.4)
Secondary or more	30.7	27.0	875 (28.3)
Wealth quintile			
Lowest	20.3	21.0	791 (20.7)
Second	20.2	19.6	759 (19.9)
Middle	13.2	22.2	696 (18.2)
Fourth	20.1	19.6	757 (19.8)
Highest	26.1	17.6	815 (21.3)

Figures do not add up to 100% because of rounding off.

### Self-reported health and functioning

Overall, more than three-quarters (76.8%) of adults rated their health as moderate or good, while few reported very good or very bad health (Table 2). On balance, men reported very good or good health more often than women (*p*<0.001). Poor self-rated health and work difficulties increased with age.

**Table 2:** Overall self-rated health status among adults aged 50 years or older, per cent distribution by Sociodemographic characteristics, South Africa, 2007–2008

	Number of respondents	Self-rated overall general health					*p*

		Very good	Good	Moderate	Bad	Very bad	
Sex							<0.001
Male	1,615	6.8	35.5	40.5	15.9	1.3	
Female	2,061	3.4	31.1	47.7	16.7	1.0	
Age group (years)							0.001
50–59	1,832	7.2	37.8	39.6	14.5	1.0	
60–69	1,126	3.1	30.7	47.4	17.6	1.3	
70–79	514	2.1	25.3	54.5	17.2	1.0	
80 +	204	1.6	22.5	48.6	24.4	3.0	
Residence							0.036
Urban	2,382	5.3	36.1	43.7	13.8	1.1	
Rural	1,293	4.2	27.3	46.0	21.2	1.2	
Marital status							<0.001
Never married	512	2.1	30.9	45.2	20.7	1.1	
Currently married	1,819	6.7	36.5	42.9	13.2	0.7	
Cohabiting	193	5.4	41.0	34.4	17.8	1.5	
Separated or divorced	217	5.0	35.1	40.4	15.1	4.4	
Widowed	867	2.6	24.6	51.1	20.6	1.2	
Education							<0.001
No formal education	967	3.5	29.1	45.9	20.0	1.4	
Less than primary	787	2.2	25.7	49.7	20.2	1.9	
Primary school completed	914	3.4	31.3	47.5	16.3	1.3	
Secondary or more	1047	9.1	43.1	37.4	9.9	0.2	
Wealth quintiles							<0.001
Lowest	755	2.6	24.4	45.2	25.4	2.5	
Second	735	3.4	32.5	45.9	16.7	1.5	
Middle	668	3.5	33.7	44.3	17.4	1.1	
Fourth	724	5.3	31.2	48.1	15.0	0.4	
Highest	778	9.5	42.8	39.6	7.9	0.3	

Figures do not add up to 100% because of rounding off.

More adults in rural (14.9%) than urban (22.4%) areas reported their health status as bad or very bad. In relation to marital status, good health was comparatively low for widowed people (*p*<0.001). There were more married people (6%) reporting very good health than those in other categories of marital status. Health status decreased with increasing age (*p*<0.001)

### Difficulty in carrying out work

Table 3 shows the self-rated difficulty in carrying out work or household activities. More than half of the men and women had at least some difficulty with work or household activities, with most (31.8% male and 36.2% female) within that group rating their difficulty as moderate. The proportion of adults with severe or extreme difficulty was generally low, although 11% of women reported having severe difficulty.

**Table 3:** Self-rated difficulty with work or household activities among adults aged 50 years or older, per cent distribution by sociodemographic characteristics, South Africa, 2007–2008

Characteristic	Number of respondents	Self-rated difficulty with work or household activities					*p*

		None	Mild	Moderate	Severe	Extreme	
Sex							<0.001
Male	1,607	42.6	17.3	31.8	7.5	0.8	
Female	2,046	34.9	16.3	36.2	11.2	1.4	
Age group (years)							<0.001
50–59	1,826	47.1	15.6	29.1	7.4	0.8	
60–69	1,116	35.1	18.4	34.6	10.5	1.5	
70 +	511	21.8	17.9	46.1	13.2	0.9	
Residence							<0.001
Urban	2,365	41.5	18.3	32.7	6.2	1.3	
Rural	1,287	32.5	13.8	37.1	15.6	0.9	
Marital status							<0.001
Never married	508	39.7	14.5	35.3	9.4	1.1	
Currently married	1,812	43.2	17.4	31.7	6.8	0.9	
Cohabiting	190	46.3	19.6	26.0	7.4	0.7	
Separated or divorced	211	31.5	16.1	33.8	12.7	5.8	
Widowed	864	27.4	15.8	41.0	15.1	0.6	
Education							<0.001
No formal education	967	33.5	17.3	35.0	12.6	0.8	
Less than primary	787	28.5	17.1	36.6	14.4	2.8	
Primary school completed	914	31.4	18.6	39.8	9.1	0.4	
Secondary or more	1047	54.1	14.4	26.7	3.1	0.5	
Wealth quintile							<0.001
Lowest	751	28.6	15.6	39.7	14.7	1.4	
Second	732	34.0	16.7	37.7	10.5	1.1	
Middle	664	37.0	19.1	31.9	9.2	2.8	
Fourth	722	39.8	16.5	33.1	10.1	0.5	
Highest	768	51.6	15.7	29.0	3.6	0.2	

Figures do not add up to 100% because of rounding off.

### Health status, QoL, and satisfaction and disability

Table 4 presents the mean Health, WHOQoL and WHODAS II scores for adults aged 50 years and above.

**Table 4:** Mean health state, WHODAS II and WHOQoL scores among adults aged 50 years or older, by sociodemographic characteristics, South Africa, 2007–2008

Characteristic	Health-state score	WHODAS II	WHOQoL

	Mean (SE)	Mean (SD)	Mean (SD)
Sex			
Male	64.5 (0.9)	18.2 (19.4)	58.3 (12.2)
Female	60.0 (0.8)	22.9 (20.4)	46.2 (12.6)
Age group (years)			
50–59	64.6 (0.9)	16.2 (16.8)	47.4 (12.4)
60–69	61.5 (1.0)	22.2 (21.1)	47.0 (12.5)
70+	57.3 (1.3)	30.3 (22.6)	46.5 (12.6)
Residence			
Urban	62.5 (0.8)	19.9 (19.3)	44.4 (12.0)
Rural	60.9 (1.!)	22.5 (21.6)	48.6 (12.5)
Education			
No formal education	58.5 (1.1.)	22.2 (20.8)	45.6 (12.3)
Less than primary	59.6 (1.1)	23.2 (20.7)	45.4 (12.3)
Primary school completed	60.4 (1.2)	23.0 (20.5)	45.2 (12.1)
Secondary or more	64.5 (1.6)	14.8 (16.8)	52.6 (11.8)
Marital status			
Never married	60.8 (1.4)	21.5 (18.6)	44.2 (12.6)
Currently married/cohabiting	64.9 (0.8)	18.1 (19.0)	49.1 (12.1)
Separated or divorced	58.4 (2.3)	23.1 (22.6)	44.2 (13.7)
Widowed	57.2 (0.9)	26.4 (21.7)	45.0 (12.4)
Wealth quintile			
Low	60.2 (1.3)	23.0 (21.4)	42.2 (11.5)
Medium	61.0 (1.4)	21.7 (20.0)	46.7 (12.1)
High	65.5 (1.5)	18.3 (18.6)	52.0 (11.6)

On average, men (64.5) had better health scores than women (60.0), as shown in Table 4. Health scores also showed predictable patterns, with health declining with increasing age and increasing with increasing levels of education. Married or cohabiting respondents had better health than those who were widowed, divorced, separated, or never married. Those in the highest wealth quintile had better health than those in the lowest wealth quintile.

Women had slightly worse evaluative well-being than men, with rural worse than urban dwellers. The overall mean score was 50.5, which implied that QoL was moderate. The scores were relatively constant over the different age groups. Clearer patterns were evident by socioeconomic status, with worse well-being reported in lower levels of wealth (55.8) and education (53.9). Currently married respondents (47.9) reported better well-being than all other marital status groups.

Table 5 presents results related to difficulties in carrying out ADLs and IADLs among adults aged 50 years or older. About two-thirds (69.9% males and 60.7% females) of older adults had no difficulties in functioning. For respondents reporting some level of difficulty with functioning, women had higher rates than men (30.9% vs. 22.3%), particularly for those with difficulties with two or more ADLs. Examining patterns by age, the proportion of people with no difficulty decreased with age. With regards to education, the proportion reporting no difficulty was higher among those with secondary education and above (70.2%) compared to those with no education (59.2%). Trends by marital status showed that those who were widowed (37.3%) had the greatest difficulty (two or more ADLs), while those who were cohabiting (17.8%) had the least. However, these results are likely to be confounded by age. The patterns found in difficulty with ADLs did not differ significantly between urban and rural areas (*p*=0.687). The mean WHODAS-II scores increased with increasing levels of ADLs deficiencies, and demonstrated good face validity. Overall, the mean disability score was 20 (out of 100), suggesting a low level of disability.

**Table 5:** Difficulty in carrying out activities of daily living (ADLs) and instrumental activities of daily living (IADLs), overall mean WHODAS score among adults aged 50 years or older, per cent distribution by sociodemographic characteristics

Characteristic	Number of activities of daily living deficiencies			Number of IADL deficiencies		
	
	0	1	2 or more	0	1	2 or more
Sex						
Male	69.9	7.8	22.3	87.4	6.8	5.8
Female	60.7	8.4	30.9	82.8	6.1	11.1
Age group (years)						
50–59	75.0	7.2	17.8	91.9	3.9	4.3
60–69	60.5	8.3	31.2	82.8	8.0	9.3
70 or more	49.9	8.7	41.4	70.2	10.5	19.3
Residence						
Urban	65.1	8.5	26.5	86.4	5.0	8.6
Rural	64.2	7.5	28.3	82.0	9.0	9.0
Education						
No formal education	59.2	8.2	32.6	81.0	6.4	12.6
Less than primary education	63.3	9.6	27.0	85.3	6.4	8.3
Primary school completed	59.7	8.7	31.6	82.3	8.7	9.0
Secondary or more	70.2	7.0	22.8	85.6	7.3	7.1
Marital status						
Never married	69.9	6.2	23.9	86.6	6.5	6.9
Currently married	68.1	8.1	23.8	88.3	5.6	6.1
Cohabiting	72.1	10.2	17.8	89.3	0.8	9.9
Separated or divorced	61.9	6.4	31.8	77.6	4.6	17.8
Widowed	53.1	9.6	37.3	77.4	9.9	12.7
Wealth quintile						
Lowest	65.9	7.8	26.4	81.0	8.4	10.5
Second	63.9	6.2	29.9	84.4	6.6	9.0
Middle	63.1	9.6	27.2	86.7	4.4	8.8
Fourth	59.5	10.5	30.1	83.3	8.2	8.5
Highest	70.7	6.4	22.9	88.5	4.5	7.0

In terms of difficulties in carrying out IADLs, older adults had more difficulty (two or more IADLs) than younger adults (29.3% vs. 4.3%); those who were less educated had more difficulty than those with higher education (12.6% vs. 7.1%); those in households with lower wealth status had more difficulty than those in higher wealth households (10.5% vs. 7.0%), and those in rural areas had slightly more difficulty than those in urban areas (9.0% vs. 8.6%); women had slightly more difficulty than men (11.1% vs. 5.8%). Looking at marital status, those who were separated or divorced (23.4%) had the most difficulty (1 or more), while those cohabiting (10.7%) had the least.

### Associations between sociodemographics, activity limitations, QoL, and poor health status

The association between poor self-rated health and the following sociodemographic factors was assessed: gender, age, marital status, race, educational level, geo-locality, and wealth status. More women reported poorer health status than their male counterparts, although this was not significant (AOR=1.30; 95% CI 0.82–2.06). Individuals in older age group reported poorer health status than the younger age group, that is, the 70+ age group was 52% more likely to do so compared to the 50–59 age group (95% CI 1.00–2.30). In terms of race, Indians were more likely to report poorer health status than all other races (AOR=4.01; 95% CI 1.27–12.70). In terms of education, those with primary education (AOR=1.83; 95% CI 1.19–2.80) and less than primary education (AOR=1.94; 95% CI 1.37–2.76) were more likely to report poorer health compared to those with secondary education. In terms of wealth status, those in low wealth quintile (AOR=2.02; 95% CI 1.14–3.57) and medium wealth quintile (AOR=1.47; 95% CI 1.01–2.13) were more likely to report poorer health status than those in the high wealth quintile. There were significant associations between marital status and locality type and self-reported poor health status (see Table 6). In terms of activity limitations, older people with moderate (AOR=3.31; 95% CI 1.82–6.03) to severe (AOR=6.67; 95% CI 2.74–16.24) IADL deficiencies were more likely to report ill health compared to those with mild IADL deficiencies. No significant associations remained for ADL in the adjusted models. In terms of QoL, individuals with low (AOR=12.78; 95% CI 7.33–22.27) to medium (AOR=3.12; 95% CI 1.24–7.81) levels of personal satisfaction (WHOQoL) were less likely to report ill-health compared to those with low WHOQoL.

**Table 6:** Multivariate analysis with poor health status

Sociodemographics	Unadjusted odds ratio (95% confidence interval=CI)	Adjusted odds ratio (95% CI) [with sociodemographics only]	Adjusted odds ratio (95% CI) [with sociodemographics, activity limitation, and quality of life]
Gender			
Female	1.00	1.00	1.00
Male	0.97 (0.77–1.22)	1.30 (0.82–2.06)	1.48 (0.85–2.57)
Age group (years)			
50–59	1.00	1.00	1.00
60–69	1.27 (0.93–1.72)	1.19 (0.91–1.55)	0.93 (0.58–1.50)
70 or more	1.42 (1.05–1.92)	1.52 (1.00–2.30)	0.93 (0.63–1.38)
Race			
White	1.00	1.00	1.00
African Black	5.90 (2.55–13.66)	2.76 (1.20–6.34)	1.52 (0.66–3.51)
Colored	2.70 (1.12–6.62)	1.46 (0.62–3.41)	0.97 (0.35–2.66)
Indian or Asian	7.51 (2.72–20.75)	4.01 (1.27–12.70)	2.13 (0.36–12.40)
Marital status			
Married	1.00	1.00	1.00
Single	1.64 (0.89–3.01)	1.51 (0.81–2.83)	1.16 (0.81–1.67)
Separated/divorced	1.44 (0.69–3.01)	0.91 (0.50–1.67)	0.80 (0.45–1.40)
Widowed	1.63 (1.23–2.17)	1.56 (1.11–2.20)	1.18 (0.78–1.80)
Educational level			
Secondary or more	1.00	1.00	1.00
Primary	2.63 (1.75–3.97)	1.83 (1.19–2.80)	1.16 (0.63–2.15)
Less than primary	3.04 (2.15–4.30)	1.94 (1.37–2.76)	1.63 (0.96–2.77)
No schooling	1.57 (0.36–6.79)	1.33 (0.34–5.27)	0.83 (0.27–2.56)
Wealth			
High	1.00	1.00	1.00
Medium	1.73 (1.25–4.05)	1.47 (1.01–2.13)	1.67 (1.03–2.71)
Low	2.25 (1.22–2.47)	2.02 (1.14–3.57)	1.27 (0.80–2.01)
Geolocality			
Rural	1.00	—	—
Urban	0.61 (0.27–1.39)		
Activity limitation and quality of life			
Activity limitation			
Mild	1.00		1.00
Moderate	4.85 (3.04–10.83)		1.29 (0.72–2.31)
Severe	9.47 (5.47–16.41)		2.22 (0.81–6.09)
Instrumental activity limitation			
Mild	1.00		1.00
Moderate	4.85 (3.04–7.74)		3.31 (1.82–6.03)
Severe	17.80 (9.63–32.87)		6.67 (2.74–16.24)
Quality of life			
High	1.00		1.00
Medium	4.39 (2.39–8.07)		3.12 (1.24–7.81)
Low	24.13 (11.71–49.76)		12.78 (7.33–22.27)

## Discussion

This study describes the health status of elderly people in South Africa. As has been the case in previous studies (11), the majority of the respondents viewed their health positively. This can be attributed to the fact that generally individuals tend to over rate their health.

As in previous studies, this study further revealed that increasing age (5, 8, 9, 11, 15, 16, 32), being female (11, 20, 32), being Black or Indian (28, 33), low education (10, 11, 32), low wealth status (10, 11, 13, 16, 32, 34), and not being married (11, 35, 36) were associated with poorer self-rated health, more difficulty in performing tasks, and lower QoL. This gap in health outcomes needs to be addressed. The SES of elderly South Africans has a definite bearing on access to quality health care services. Elderly people with low SES depend mainly on public sector health services for their daily needs, but the health system is not appropriately designed to provide them with secure health services. South Africa has pledged action to address the needs and well-being of older persons through the African Union Africa Health Strategy 2007–2013 (37). The challenge is to overcome the policy inaction and research inadequacies (38, 39).

Similar to other studies (14, 32), increasing levels of deficiencies in ADLs and IADLs and lower QoL were associated with greater odds of reporting poorer health status among the elderly. The health, disability, and living conditions in old age are policy concerns throughout the world. The deterioration of health status of older people with increasing age will induce greater demand for long-term care. Therefore, the South African government needs to predict and prepare for increasing demand for health services for age-related health conditions. It should be noted that although the study outcome is based on self-rated data, which are prone to bias, the self-reported measures, which were used in this study, were validated. Further, in spite of self-rated health being a subjective measure of one's health, it has been found to be a good measure of complex health problems (32, 40). It is referred to as a good predictor for numerous health-related outcomes (41).

However, follow-up surveys are needed to monitor trends and patterns over time. The cross-sectional nature of SAGE Wave 1 does not permit an investigation of the cause–effect relationship between self-reported health and the independent variables. Thus, follow-up SAGE surveys are planned to continue to monitor trends on the health status and well-being of elderly people and how health and social policies can impact on them. Planned follow-up SAGE surveys will be repeated 2–3 times in 5–10 years, and, based on this, it is anticipated that policies and programs will be further refined.

This population-based study has generated generalizable estimates of the health status of the older people in South Africa. It is clear from the foregoing discussion that the health status of older South Africans deteriorates with increasing age. This evidence not only contributes to bridging the gap in knowledge in as far as the health status of elderly people in South Africa is concerned, but it also provides credence for the actions that are required to address the needs of all elderly South Africans.

The evidence generated by this study can be used to inform national health and social development policies to mainstream economic, social, and physical support for the elderly in South African and elsewhere.
